# A Scalable, Multi-format Approach to Polypharmacy Education in Rural Older Adults: An Exploratory Pilot Study Comparing Self-Paced Packet Versus In-Person Education Interventions

**DOI:** 10.7759/cureus.91101

**Published:** 2025-08-27

**Authors:** Caleb Zimmerman, Aditya Shah, Patrick Fakhoury, Christya Haddad, Keira Tolmie, Erin Williams, Jyotsna Pandey

**Affiliations:** 1 College of Medicine, Central Michigan University, Mt. Pleasant, USA; 2 College of Science and Engineering, Central Michigan University, Mt. Pleasant, USA

**Keywords:** health education & information, medication management, older adults and rural community, polypharmacy, rural health challenges

## Abstract

Background

Polypharmacy poses significant risks for older adults, especially in rural areas where healthcare access may be limited. This exploratory pilot study assessed the feasibility and potential impact of two educational interventions aimed at supporting medication management among rural older adults.

Methodology

We conducted a comparative intervention study from July 2024 to October 2024. Rural adults aged ≥60 years taking medications participated in in-person sessions (n = 18) or received self-paced packets (n = 33). Both interventions used the WHO’s Five Moments for Medication Safety framework. Pre- and post-surveys assessed knowledge, confidence, and medication management practices using REDCap. Statistical analysis included descriptive statistics and inferential testing to examine associations between polypharmacy status and medication management outcomes.

Results

Both interventions improved polypharmacy knowledge and management confidence. Inferential analysis revealed significant associations validating intervention targets: polypharmacy (>4 medications) was significantly associated with medication management difficulty (p = 0.011) and multiple simultaneous challenges (p = 0.024, odds ratio = 9.99). In-person participants’ recognition of polypharmacy risks increased from 50% to 77.8%, while provider communication confidence rose from 88.9% to 94.4%. The self-paced packet achieved wider reach (94.4% more participants), with 100% reporting information was clear and relevant, and 93.9% feeling more confident discussing medications with providers. Groups differed significantly in age distribution (p = 0.002) but not in polypharmacy burden (p = 0.766). The packet format extended access by addressing barriers such as transportation, technology access, and mobility limitations, reaching 96.8% of participants outside the primary study county compared to 47.1% for in-person sessions.

Conclusions

Both interventions improved outcomes, but the self-paced packet demonstrated superior scalability and engagement. Statistical analysis confirmed that polypharmacy creates significant management challenges, validating targeted educational interventions. The packet’s multi-format design (printed materials, USB drives, QR codes, web links) effectively reduced participation barriers and promoted equitable access, offering a promising model for rural health interventions.

## Introduction

Polypharmacy, commonly defined as the concurrent use of five or more medications [1], is a well-documented risk factor for adverse drug reactions, medication non-adherence, falls, and impaired physical and cognitive functioning among older adults [2-6]. In this study, we operationally define polypharmacy as the use of more than four medications for analytical purposes, which is consistent with the conventional definition of five or more medications. Individuals taking five or more medications face an 88% higher risk of experiencing an adverse drug reaction compared with those taking fewer than five medications, making them more likely to be hospitalized [7,8]. Research indicates that these risks are particularly pronounced in rural settings, where healthcare access is limited and care is often fragmented [9].

Multiple studies have highlighted the widespread nature of polypharmacy among older adults. Research consistently documents that over 50% of older adults in the United States regularly take five or more medications, with this pattern observed across diverse older adult populations [10-12]. Older adults often lack adequate knowledge of medication safety, with many unaware of drug interaction risks and potential side effects [13].

Education-based interventions aimed at reducing polypharmacy risks have demonstrated varying degrees of success. Clinical pharmacist interventions for reducing medication errors in older adults have been studied, yet overall evidence for medication error reduction strategies remains weak [14]. While educational interventions for older adults have shown effectiveness in improving health knowledge and confidence, research indicates these interventions face implementation challenges that can limit their reach and effectiveness [15,16].

This exploratory study compared two medication management interventions aimed at improving medication management practices among rural older adults. The first intervention included traditional in-person educational sessions, and the second intervention was a self-paced multi-format information packet. Both approaches employed the same educational tools to maintain consistency while accommodating different accessibility needs and preferences. We hypothesized that both interventions would improve medication knowledge and healthcare provider communication confidence, while the self-paced packet would demonstrate superior reach and accessibility due to its ability to address common rural barriers such as transportation challenges, limited technology access, and mobility limitations that often prevent participation in traditional in-person programming. This research aims to ultimately enhance polypharmacy knowledge, healthcare provider communication confidence, and medication management practices among rural older adults.

## Materials and methods

We conducted this comparative intervention study from July 2024 to October 2024. Two educational interventions for improving polypharmacy management among rural older adults were employed: an in-person educational session and a self-paced packet. In-person presentations occurred from July 2024 to September 2024, while self-paced packet distribution took place from September 2024 to October 2024.

Participants were recruited from rural areas in Michigan through multiple outreach methods, including local senior centers, libraries, community groups, religious institutions, and Commission on Aging offices. Promotional strategies encompassed flyers, emails, phone calls, and in-person visits.

Inclusion and exclusion criteria

Eligibility criteria included being 60 years of age or older, residing in a rural area (as defined by the Health Resources and Services Administration using the Rural Health Grants Eligibility Analyzer), and currently taking at least one medication regularly [17]. Medications could include prescription drugs, over-the-counter medications, or supplements. While the project focused on polypharmacy, participants taking any number of medications were eligible, as the goal of our study was to improve medication management among older adults in rural areas. Participants were also required to demonstrate sufficient cognitive capacity to provide informed consent and independently complete the surveys. Cognitive ability was assessed informally during enrollment interactions based on participants’ ability to understand study information, ask relevant questions, and communicate effectively. No formal cognitive screening tools were used.

For this pilot study, we selected rural Michigan as our target area due to documented healthcare access challenges and limited polypharmacy education resources in these communities. We partnered with local senior centers, libraries, community groups, religious institutions, and Commission on Aging offices because they serve as trusted community hubs with established relationships with older adults and existing infrastructure for health programming. We determined the sample size based on feasibility rather than formal power calculations. We aimed to recruit 15-20 participants for the in-person intervention based on typical community center capacity and 30-40 participants for the self-paced packet intervention to assess scalability. We did not conduct pre-study power calculations as this was an exploratory pilot study designed to inform future larger-scale research.

Institutional review board determination

The Central Michigan University Institutional Review Board (IRB) reviewed this study (protocol: #2023-1003) and determined it to be exempt from federal IRB review requirements under 45 CFR 46.104(d) categories 2(ii) and 3(i)(B). All participants provided informed consent before participation.

Intervention design

Following eligibility screening, participants were assigned to either the in-person or self-paced packet intervention format. Both formats were designed to enhance participants’ awareness of polypharmacy and its associated risks, increase their confidence, and support effective medication management. The interventions were structured around the WHO’s Five Moments for Medication Safety framework [18] and included the Drug List Collection Tool.

Participant knowledge, confidence, and medication management practices were assessed using project-developed surveys administered before and after the intervention. In this study, medication knowledge refers to participants’ understanding of the purpose of each medication they are currently taking, while polypharmacy knowledge refers to their understanding of the definition of polypharmacy. The pre-survey collected demographic information, current medication practices, confidence in communicating with healthcare providers, and knowledge of polypharmacy risks. The post-survey measured changes in these areas and gathered feedback on the usefulness, clarity, and relevance of the intervention materials.

Both formats included the Five Moments for Medication Safety framework, which provides a structured approach to critical points in medication use, as well as the Drug List Collection Tool, which helps participants record and organize their medications with details such as dosage, frequency, and potential side effects.

In-Person Intervention

This consisted of a single 60-minute educational session conducted at local community centers or senior meeting spaces. Participants first completed a pre-survey at the beginning of the session. The core of the session was a researcher-led presentation focused on increasing awareness of polypharmacy, emphasizing its associated risks, and highlighting the importance of medication safety. Key topics included an overview of polypharmacy and why it matters for older adults; the Five Moments for Medication Safety, presented through real-world scenarios and paired with reflective questions [18]; hands-on training with the Drug List Collection Tool, during which participants received printed templates and guidance on documenting their medications; and a Q&A with peer discussion to encourage engagement, share experiences, and address common challenges. Immediately after the presentation, participants completed a post-survey. Printed materials were also provided for future reference.

Self-Paced Packet Intervention

This format was developed to address common barriers to in-person participation, such as transportation challenges, cost, and limited accessibility. Each packet included several components designed to support independent learning. The educational materials featured a printed guide that explained the Five Moments for Medication Safety [18], using real-life examples and visual aids to enhance understanding. Step-by-step instructions were also provided to help participants use the Drug List Collection Tool effectively. To accommodate varying levels of internet access, the packet contained digital resources in two formats, namely, a USB drive with a recording of the educational presentation used in the in-person sessions, and QR codes along with web links for participants who preferred to view the materials online. Finally, the packet included printed pre- and post-surveys, along with pre-addressed, stamped return envelopes, making it easy for participants to complete and return the surveys even if they preferred paper-based formats or lacked internet access.

Statistical methods

We analyzed quantitative data using R statistical software (version 4.3.0) and REDCap for data collection and management. Our analysis approach included both descriptive and inferential statistics to comprehensively evaluate intervention effectiveness and explore meaningful associations within baseline data.

Descriptive statistics, including frequencies, percentages, means, and medians, were calculated to characterize participant demographics, medication patterns, and baseline health conditions. Pre-post intervention changes were assessed using percentage point differences.

For inferential statistical testing, we conducted hypothesis-driven analyses organized into the following three categories: within-group associations for each intervention type, and between-group comparisons. Fisher’s exact tests were used for categorical variables when cell counts were small, particularly for examining associations between polypharmacy status and medication management outcomes. Chi-square tests were applied to compare demographic characteristics between intervention groups. Spearman’s rank correlation examined relationships between continuous and ordinal variables, while Kruskal-Wallis tests compared multiple groups with ordinal outcomes.

Statistical significance was set at an alpha of 0.05 for all tests. Given the exploratory nature of this pilot study, we did not adjust for multiple comparisons but report all p-values transparently. Effect sizes are reported where appropriate, including odds ratios (ORs) for Fisher’s exact tests and correlation coefficients for associations. Sample sizes were determined by practical recruitment constraints rather than formal power calculations, which limits our ability to detect small effects but provides valuable pilot data for future larger studies.

Ethical considerations

We maintained confidentiality through the de-identification of survey data before analysis. We designed materials and surveys for accessibility, including considerations for literacy levels and visual impairments.

## Results

The primary study aim was to evaluate two educational intervention formats, namely, in-person educational sessions and self-paced packets, for improving polypharmacy knowledge, healthcare provider communication confidence, and medication management practices among rural older adults. Secondary aims included assessing intervention accessibility, participant engagement, communication confidence, and participants’ intent to implement medication safety strategies. Additionally, we conducted inferential statistical analyses to examine associations between polypharmacy status and medication management outcomes within each intervention group, explore relationships between demographic factors and medication-related behaviors, and compare baseline characteristics between intervention groups to understand potential differences in recruitment patterns and population reach. These outcomes were measured through pre- and post-intervention surveys.

Study participation and response rates

**Figure 1:**
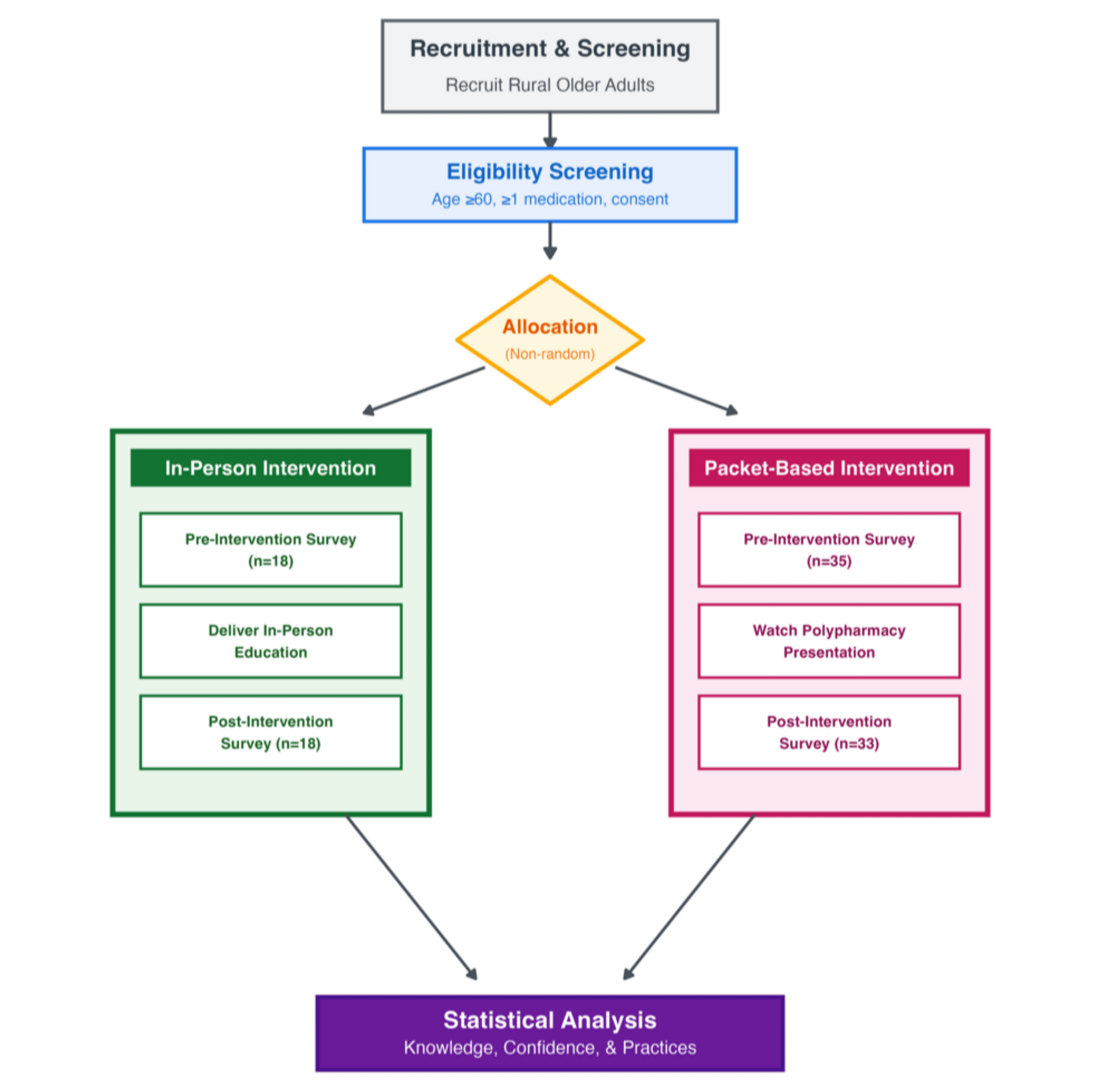
Study flow diagram. This flowchart illustrates the recruitment, allocation, intervention delivery, and assessment process for both the in-person arm (n = 18) and self-paced packet arm (n = 35 to 33) of the study. The diagram shows participant progression through each phase of the intervention, from initial recruitment and screening to data analysis of outcomes related to knowledge, confidence, and medication management practices.

A total of 53 rural older adults participated across both intervention groups. The in-person intervention achieved a 100% completion rate (18/18 participants completed both pre- and post-surveys). The self-paced packet intervention achieved a 94.3% completion rate (33/35 participants who completed pre-surveys also completed post-surveys, representing a 5.7% attrition rate). The two participants who did not return post-surveys did so voluntarily, with no follow-up contact attempted per study protocol to respect participant autonomy (Figure 1).

Participant characteristics

Demographic Characteristics

Notable demographic differences existed between the intervention groups. In-person intervention participants (n = 18) had a mean age distribution, with 33.3% aged 70-79 years, 16.7% aged 60-69 years, 38.9% aged 80-89 years, and 11.1% aged 90+ years, representing an older population overall. Gender distribution showed 61.1% female and 38.9% male participants. Geographic distribution indicated 52.9% resided in Isabella County, with 47.1% from surrounding rural counties. All participants (100%) reported English as their primary language. Self-paced packet participants (n = 35) showed a younger age distribution with 57.1% aged 70-79 years, 37.1% aged 60-69 years, 2.9% aged 80-89 years, and 2.9% aged 90+ years. Gender distribution was similar with 57.1% female, 40.0% male, and 2.9% who put other as gender. Geographic distribution revealed substantially broader rural reach, with 96.8% residing outside Isabella County compared to only 47.1% in the in-person group. All participants (100%) reported English as their primary language.

Medication Use Patterns

Both groups demonstrated high polypharmacy prevalence, though measured using different thresholds. In-person participants showed 66.6% taking ≥six medications daily (33.3% taking 10+ medications, 33.3% taking six to nine medications), 5.6% taking three to five medications, and 27.8% taking one to two medications daily. Self-paced packet participants showed 58.8% taking more than four medications daily, 29.4% taking two to four medications daily, and 11.8% taking one medication daily. Despite the different measurement approaches, both groups represented populations with substantial medication management needs.

Health Condition Prevalence

In-person participants most commonly reported hypertension (50.0%, n = 9/18), with additional medication management challenges including understanding medication purpose (50.0%) and remembering medication timing (31.3%). Self-paced packet participants reported similar patterns, with hypertension being the most common (45.2%, n = 14/31 respondents), followed by gastrointestinal issues (25.8%), depression/anxiety (25.8%), and thyroid disorders (16.1%). The majority (61.3%) reported additional health conditions beyond the provided categories. Response rate for health conditions was 88.6% (31/35 participants). These health profiles indicate that both groups had complex medical needs requiring multiple medications.

Educational Background

Self-paced packet participants revealed significant unmet educational needs, with 60.0% (n = 21/35) reporting they had not previously received formal medication management education. This baseline measure was not collected in the in-person group, but suggests substantial gaps in medication education access among rural older adults.

Inferential statistical findings

Packet Group Internal Associations

**Figure 2:**
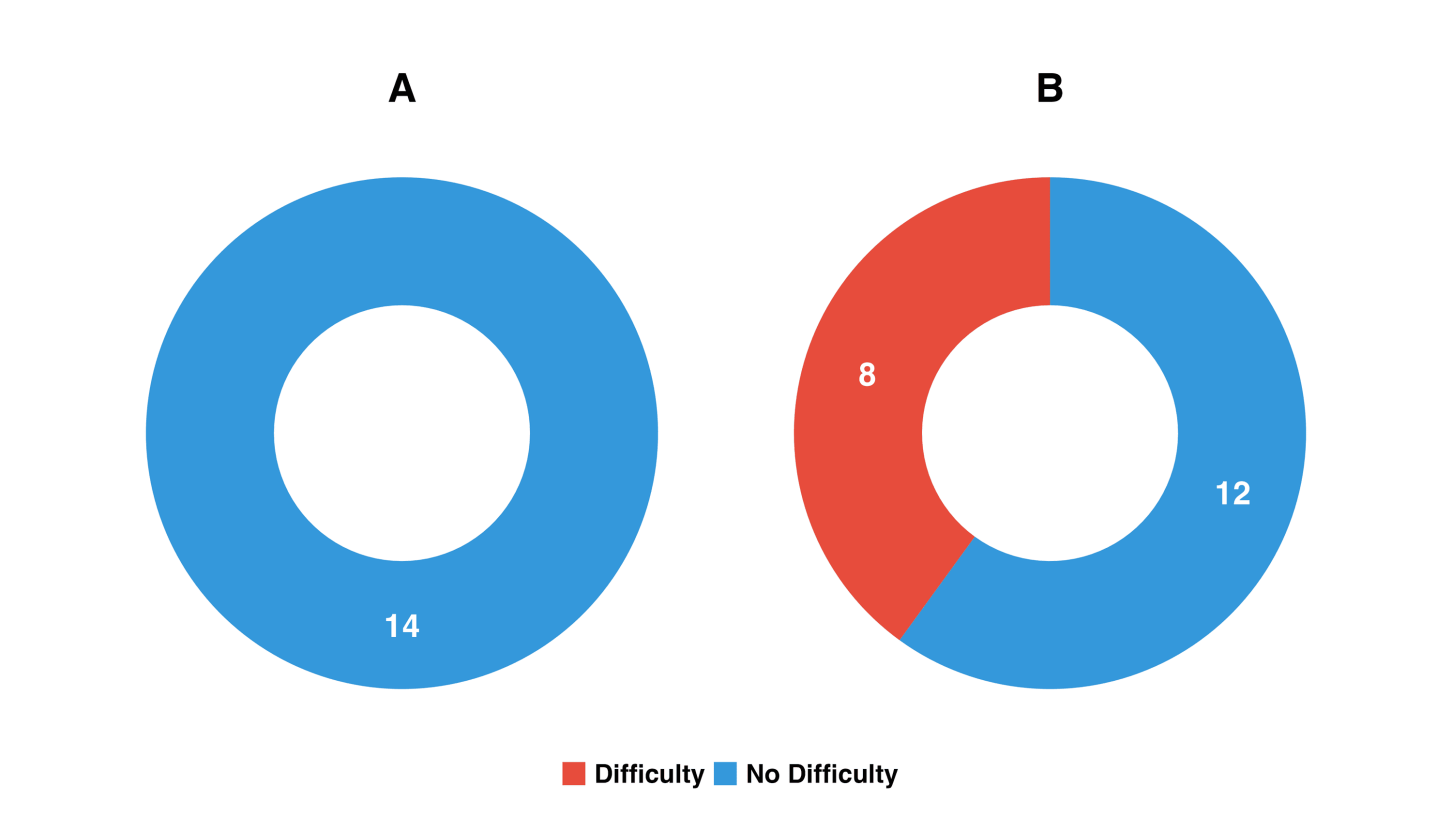
Medication management difficulty by polypharmacy status. This figure illustrates the association between polypharmacy status and reported difficulty with medication management among participants in the packet group. Panel A shows responses from non-polypharmacy participants (≤4 medications), all of whom reported no difficulty (n = 14). Panel B shows responses from polypharmacy participants (>4 medications), among whom 40% (8 out of 20) reported difficulty. Medication management difficulty is indicated by color: red for “Difficulty” and blue for “No Difficulty.” Fisher’s exact test indicated a significant association between polypharmacy and difficulty (p = 0.011).

Within the packet intervention group, we identified several significant associations that highlight medication management challenges among rural older adults. Most notably, participants taking more than four medications were significantly more likely to report medication management difficulties compared to those taking four or fewer medications (Fisher’s exact test, p = 0.011). The analysis revealed that none of the fourteen participants with non-polypharmacy status reported management difficulties, while eight of the twenty participants with polypharmacy status reported such challenges (Figure 2).

**Figure 3:**
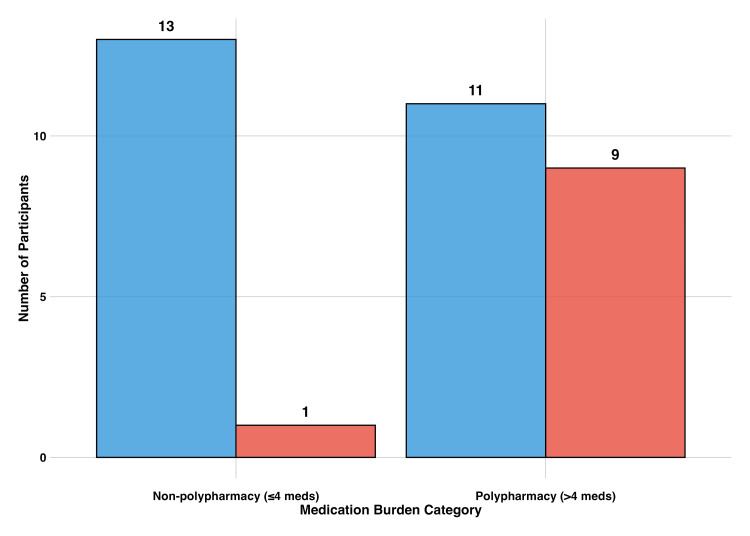
Challenge burden by polypharmacy status. This figure illustrates the relationship between polypharmacy status and cumulative medication challenges. Participants with polypharmacy (>4 medications) were significantly more likely to experience multiple simultaneous challenges compared to those without polypharmacy (≤4 medications) (Fisher’s exact test, p = 0.024, OR = 9.99). Nearly half (9/20) of polypharmacy participants experienced multiple challenges, versus only one participant (1/14) in the non-polypharmacy group. Challenge level is indicated by color: few challenges (blue) and multiple challenges (red).

Additionally, polypharmacy status significantly predicted experiencing multiple medication challenges simultaneously (Fisher’s exact test, p = 0.024, OR = 9.99). This finding suggests that medication burden creates compounding management challenges rather than isolated problems. Among participants with non-polypharmacy, thirteen reported few challenges, while only one reported multiple challenges. In contrast, among those with polypharmacy, eleven reported few challenges while nine reported multiple challenges (Figure 3).

Other associations within the packet group were not statistically significant. Age did not significantly influence provider communication confidence, with older adults (80+) reporting similar confidence levels as younger participants (60-79) (Fisher’s exact test, p = 1.0). Prior medication education experience was not significantly associated with current medication knowledge (Fisher’s exact test, p = 0.259), though this may reflect ceiling effects given high baseline knowledge levels. Gender differences in medication management confidence were also non-significant (Fisher’s exact test, p = 0.672).

In-Person Group Internal Associations

The in-person intervention group showed different patterns of associations. No significant correlation was found between medication count and provider communication confidence (Spearman’s ρ = -0.104, p = 0.680). This suggests that in this group, taking more medications did not diminish confidence in communicating with healthcare providers.

Age was not significantly associated with medication knowledge levels across the four age groups in this sample (Kruskal-Wallis H = 3.492, p = 0.322). Descriptive analysis showed that the 80-89-year age group had the highest median medication knowledge, while other groups showed more variable patterns. Additionally, polypharmacy knowledge did not differ significantly by age (Fisher’s exact test, p = 0.471), with both older (80+) and younger (60-79) participants showing similar patterns of correct versus incorrect responses.

Between-Group Comparisons

**Figure 4:**
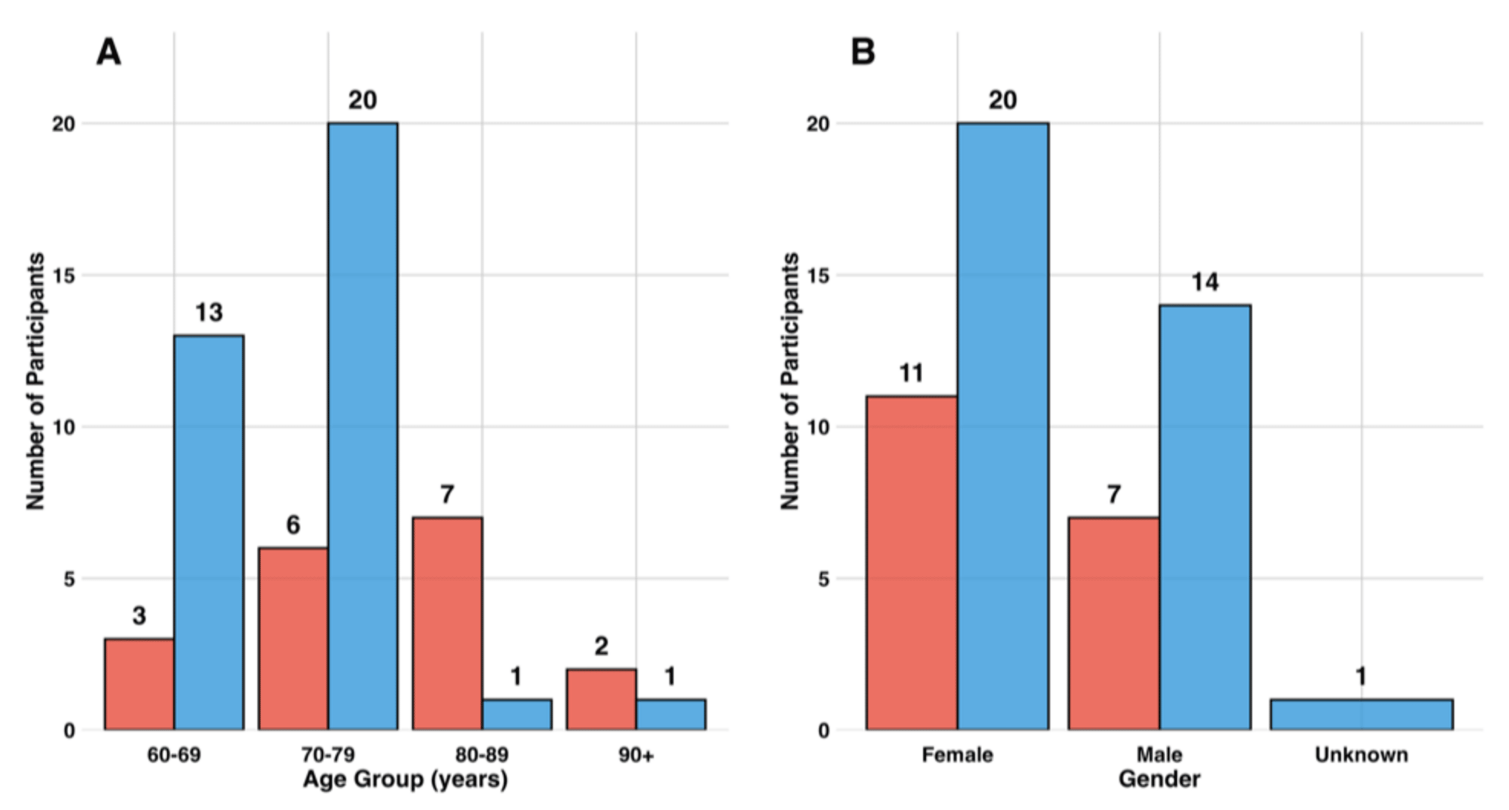
Age and gender distribution by intervention group. Panels A and B display demographic characteristics of participants across the two intervention groups: in-person (red) and packet (blue). Panel A shows age distribution, with a significant difference between groups (χ² = 14.679, p = 0.002). Panel B shows gender distribution, with no significant difference (χ² = 0, p = 1.000). The in-person group had proportionally more participants aged 80 years and older, while both groups exhibited similar gender distributions, with approximately 60% female participation.

Significant demographic differences existed between the two intervention groups that are important for interpreting comparative outcomes. Age distribution differed significantly between groups (χ² = 14.679, df = 3, p = 0.002), with the in-person group having proportionally more participants aged 80 years and older compared to the packet group. Specifically, the in-person group included 50% of participants aged 80+, while the packet group included only 5.8% in this age range. Despite the age differences, gender distribution was nearly identical between groups (χ² = 0, df = 1, p = 1.0), with both groups showing approximately 60% female and 40% male participation. Importantly, polypharmacy burden did not differ significantly between groups (Fisher’s exact test, p = 0.766), indicating that both interventions reached populations with similar medication complexity despite their different recruitment and delivery methods. This similarity in polypharmacy prevalence supports the validity of comparing intervention effects across formats, despite demographic differences such as age (Figure 4).

Our comprehensive inferential statistical analysis examined associations within each intervention group and compared baseline characteristics between groups. The analysis was organized into the following three main categories: within-group associations for the packet intervention, within-group associations for the in-person intervention, and between-group demographic comparisons. Within the packet group, we identified two statistically significant associations that validate our intervention targets. Most notably, participants with polypharmacy (more than four medications) were significantly more likely to experience medication management difficulties compared to those with fewer medications (Fisher’s exact test, p = 0.011). Additionally, polypharmacy status significantly predicted experiencing multiple medication challenges simultaneously (Fisher’s exact test, p = 0.024, OR = 9.99), suggesting that medication burden creates compounding rather than isolated problems. In contrast, the in-person group showed no significant correlations between medication count and provider communication confidence (Spearman’s ρ = -0.104, p = 0.680), and age was not associated with medication knowledge levels (Kruskal-Wallis H = 3.492, p = 0.322). Between-group comparisons revealed significant age differences (χ² = 14.679, p = 0.002) but similar gender distribution and polypharmacy burden, validating our ability to compare intervention effects across delivery methods (Table 1).

**Table 1:** Inferential statistical analysis results. This table presents all statistical tests conducted, grouped by analysis category (between-group, in-person group, packet group). Reported results include test statistics, p-values, effect sizes, and interpretations. Three statistically significant findings emerged: groups differed in age distribution (p = 0.002), polypharmacy was associated with greater management difficulty (p = 0.011), and polypharmacy predicted multiple challenges (p = 0.024). Statistical significance is denoted by asterisks.

Analysis category	Statistical test	Test statistic	P-value	Interpretation
Between-group comparisons				
Age distribution	Chi-square test	χ² = 14.679	0.002**	Groups differ significantly in age
Gender distribution	Chi-square test	χ² = 0	1.000	Groups have a similar gender distribution
Polypharmacy burden	Fisher’s Exact test	OR = 1.39	0.766	Similar polypharmacy burden between groups
In-person group associations				
Medication count ↔ Provider communication confidence	Spearman correlation	ρ = -0.104	0.680	No correlation between medication count and provider communication confidence
Age → Medication knowledge	Kruskal-Wallis test	H = 3.492	0.322	No age effect on medication knowledge
Age ↔ Polypharmacy knowledge	Fisher’s Exact test	OR = Inf	0.471	No age differences in polypharmacy knowledge
Packet group associations				
Polypharmacy → Management difficulty	Fisher’s Exact test	OR = undefined	0.011**	Polypharmacy significantly increases difficulty
Age → Provider communication confidence	Fisher’s Exact test	OR = 1.52	1.000	No age effect on provider communication confidence
Prior education → Medication knowledge	Fisher’s Exact test	OR = Inf	0.259	Prior education not associated with medical knowledge
Gender → Management confidence	Fisher’s Exact test	OR = 1.96	0.672	No gender differences in confidence
Polypharmacy → Multiple challenges	Fisher’s Exact test	OR = 9.99	0.024**	Polypharmacy predicts multiple challenges

Primary outcomes: knowledge and understanding with statistical context

Polypharmacy Knowledge and Understanding Changes

For the packet intervention, the definition of polypharmacy (polypharmacy knowledge) was assessed in both the pre- and post-surveys. Correct identification increased from 94.3% to 100%. In the in-person intervention, only 11.8% of participants selected the correct definition in the pre-survey. Because the survey formats differed (packet participants answered a True/False item, while in-person participants completed a multiple-choice item with distractors), we did not conduct inferential testing and instead report descriptive statistics to illustrate change. In the in-person group, change over time was assessed with an item included in both pre- and post-surveys that addressed risk recognition. Recognition that polypharmacy management minimizes drug interaction risks increased from 50.0% to 77.8% (a +27.8 percentage point change).

Healthcare Provider Communication Outcomes

**Figure 5:**
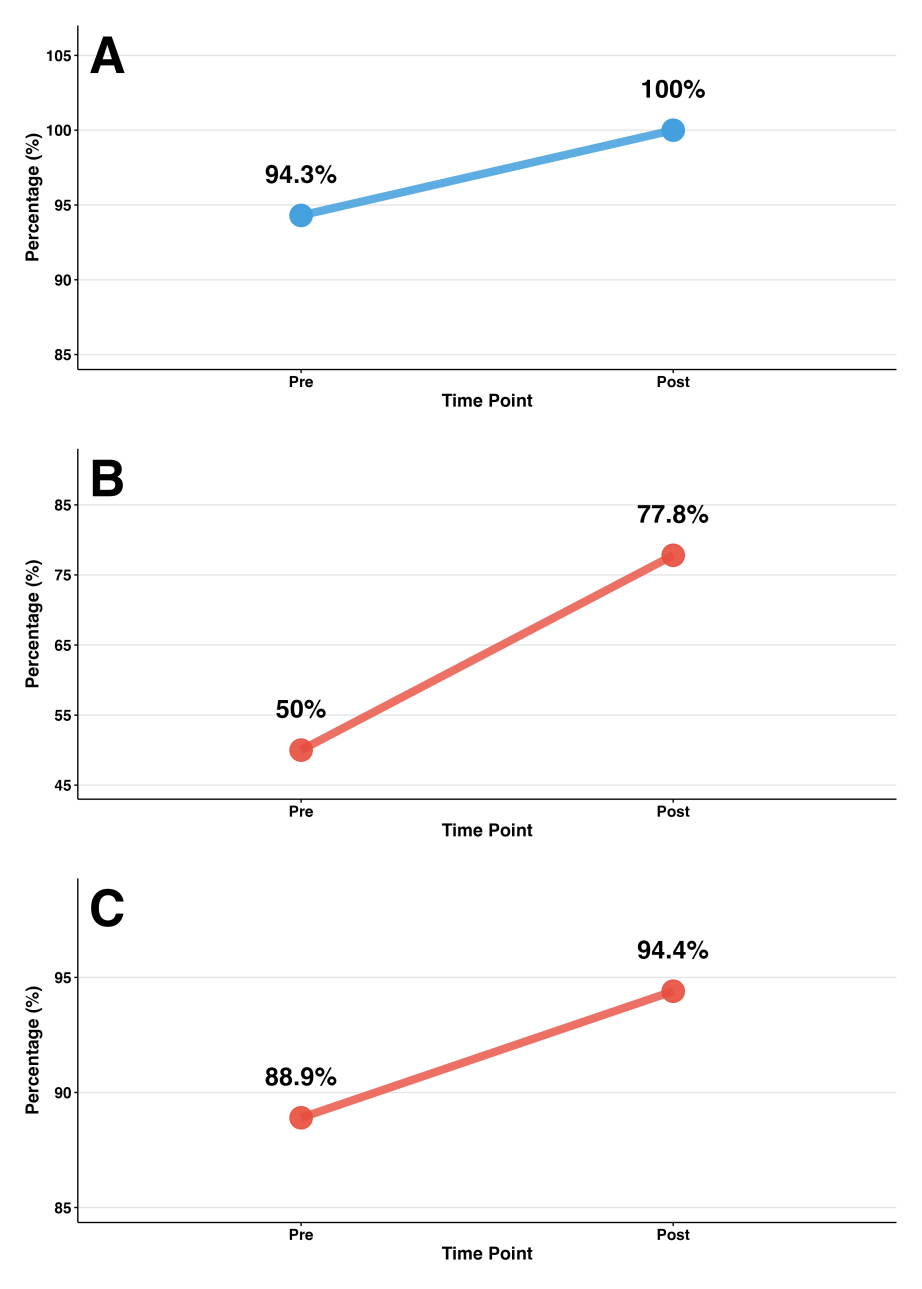
Pre-post intervention knowledge and confidence changes. This figure displays pre–post intervention changes in knowledge and confidence outcomes by group, with each panel representing a specific outcome. Panel A shows an increase in polypharmacy knowledge within the packet group, from 94.3% at baseline to 100% post-intervention. Panel B shows a 27.8 percentage point improvement in polypharmacy risk recognition in the in-person group (from 50% to 77.8%), and Panel C shows a 5.5 percentage point improvement in provider communication confidence (from 88.9% to 94.4%). Lines connect matched pre- and post-intervention responses. Intervention groups are color-coded: packet (blue) and in-person (red).

Both interventions showed improvements in provider communication confidence, though measured differently. The in-person group showed a 5.5 percentage point increase in participants feeling confident asking healthcare providers medication questions (88.9% to 94.4%). The packet group achieved 93.9% agreement that they felt more confident discussing medications with providers after the intervention (Figure 5).

Secondary outcomes: accessibility, communication confidence, and implementation intent

Intervention Accessibility and Reach

The self-paced packet intervention demonstrated superior reach, recruiting 94.4% more participants than in-person sessions (35 vs. 18 participants). Geographic accessibility was enhanced, with 96.8% of packet participants residing outside the primary study county compared to 47.1% of in-person participants, indicating improved access for geographically isolated rural residents.

Implementation Intent

In-person intervention achieved 83.3% (n = 15/18) implementation intent, while the self-paced packet intervention achieved 100% (n = 33/33) agreement for planning to use discussed strategies.

Intervention Clarity and Relevance

Both formats received positive feedback regarding clarity and relevance. The in-person intervention achieved 72.2% (n = 13/18) rating information as “very clear” and 72.2% rating it as “very relevant” to personal medication management. The self-paced packet intervention achieved 100% (n = 33/33) agreement that information was clear and relevant.

These findings demonstrate that both intervention formats can effectively improve medication-related knowledge and communication confidence, while our inferential analysis reveals that polypharmacy creates significant management challenges that justify targeted educational interventions. The significant associations between medication burden and management difficulties validate the clinical importance of addressing polypharmacy in this population, while the lack of significant between-group differences in medication burden suggests both interventions reached appropriate target populations.

## Discussion

This exploratory pilot study aimed to assess the feasibility and potential impact of two educational intervention formats for enhancing polypharmacy knowledge, healthcare provider communication confidence, and medication management practices among rural older adults. Both interventions demonstrated promising trends toward achieving these goals, though with varying levels of feasibility, reach, and potential effectiveness. The in-person intervention demonstrated a 27.8 percentage point improvement in polypharmacy risk recognition and maintained high baseline confidence levels in provider communication. The self-paced packet intervention achieved broader geographic reach by enabling distribution beyond Isabella County (96.8% of packet participants vs. 47.1% of in-person participants resided outside Isabella County) while also recruiting 94.4% more total participants and achieving unanimous positive response rates across all measured outcomes.

Within the packet intervention group, participants with polypharmacy (more than four medications) were significantly more likely to experience medication management difficulties (Fisher’s exact test, p = 0.011), as well as multiple medication challenges simultaneously (Fisher’s exact test, p = 0.024, OR = 9.99). This finding demonstrates that medication burden creates compounding management difficulties rather than isolated problems. Medication adherence is already a challenge for the elderly population, likely due to the cognitive burden that polypharmacy places on individuals, especially those with memory issues or limited health literacy [15]. As the number of medications increases, the complexity of medication adherence for older adults will also increase.

Interestingly, other hypothesized associations within the packet group were not statistically significant. Age did not significantly influence provider communication confidence (Fisher’s exact test, p = 1.0), suggesting that older adults aged >65 years reported similar levels of confidence in communicating with healthcare providers regardless of their specific age. Prior medication education experience was not significantly associated with current medication knowledge (Fisher’s exact test, p = 0.259), though this may reflect ceiling effects given the high baseline knowledge levels in this population. Gender differences in medication management confidence were also non-significant (Fisher’s exact test, p = 0.672), indicating that male and female participants reported similar levels of confidence in managing their medications. This aligns with prior findings showing that antihypertensive medication adherence did not significantly differ between men and women [19].

In the in-person intervention group, no significant correlation was observed between medication count and provider communication confidence (Spearman’s ρ = -0.104, p = 0.680), suggesting that participants maintained confidence in communicating with providers regardless of their medication burden. Additionally, age was not significantly associated with medication knowledge (Kruskal-Wallis H = 3.492, p = 0.322), and polypharmacy knowledge did not significantly differ by age (Fisher’s exact test, p = 0.471).

Between-group comparisons revealed important demographic differences that have implications for intervention delivery and interpretation. Age distribution differed significantly between groups (χ² = 14.679, df = 3, p = 0.002), with the in-person group having proportionally more participants aged 80 years and older (50% vs. 5.9% in the packet group). This age difference likely reflects our recruitment strategy, as in-person sessions were conducted at locations that serve older adults with higher care needs, including PACE (Program for All-Inclusive Care for the Elderly) programs, senior community centers, and assisted living facilities where participants may have greater mobility limitations or health complexities. In contrast, the packet intervention reached a broader geographic area and may have attracted younger, more independent rural older adults who were able to participate through mail-based distribution.

Despite the significant age differences, gender distribution was nearly identical between groups (χ² = 0, df = 1, p = 1.0), and importantly, polypharmacy burden did not differ significantly between groups (Fisher’s exact test, p = 0.766). This similarity in medication complexity validates our ability to compare intervention effects across formats and suggests that both delivery methods successfully reached populations with substantial medication management needs.

Our findings align with previous research demonstrating the effectiveness of structured medication education interventions [20]. While some evidence supports clinical pharmacist interventions for reducing medication errors in older adults, the overall evidence for such strategies remains limited [14]. Similarly, our in-person intervention improved medication knowledge but faced accessibility barriers. In contrast, the self-paced packet approach addressed these limitations while maintaining educational effectiveness.

The baseline polypharmacy prevalence in our sample (66.6% taking ≥six medications in the in-person group, 58.8% taking more than four medications in the packet group) exceeds the national average, which reports that approximately 50% of older adults take five or more medications [10]. This higher prevalence likely reflects the rural setting and recruitment through senior centers, suggesting our sample represented individuals with greater medication management needs. The significant associations we identified between polypharmacy and management difficulties (p = 0.011) and multiple challenges (p = 0.024) provide empirical support for the clinical importance of addressing medication burden in this population.

Several unexpected findings emerged from our statistical analysis. The unanimous positive response (100%) across multiple measures in the self-paced packet group was surprising and may reflect response bias inherent in self-administered surveys. This contrasts with the more varied responses in the in-person group, where direct researcher administration may have elicited more honest feedback. The substantial difference in baseline polypharmacy definition recognition between groups (11.8% for the in-person intervention and 94.3% for the self-paced packet intervention) exceeded expectations. This discrepancy was at least partly due to differences in question format, as the in-person group received a multiple-choice question with several distractors, while the packet group was asked a simpler true or false question. Additionally, 60% of packet participants had no prior medication education, a concerning finding that may be due to barriers in healthcare access, limited outreach, or a lack of tailored programs in rural settings.

The lack of significant associations in some anticipated areas provides important insights. For instance, the non-significant relationship between age and provider communication confidence in the packet group (p = 1.0) and the non-significant relationship between medication count and provider communication confidence in the in-person group (p = 0.680) suggest that, within this sample, neither older age nor higher medication burden was a barrier to feeling confident when communicating with healthcare providers. Similarly, the non-significant association between prior medication education and current medication knowledge (p = 0.259) may indicate that previous educational approaches were either ineffective or that current high knowledge levels represent a ceiling effect.

This study’s primary strength lies in its comparative design addressing real-world accessibility barriers facing rural older adults, supported by robust statistical analysis that validates key hypotheses about polypharmacy challenges. The multi-format packet approach (printed materials, USB drives, QR codes, web links) represents an innovative solution to digital literacy and connectivity challenges. Additional strengths include high completion rates (100% in-person, 94.3% packet), diverse geographic reach across rural Michigan counties, practical application of WHO evidence-based frameworks, and comprehensive inferential statistical testing that provides evidence for clinical significance beyond descriptive trends.

These findings offer meaningful insight for rural health programming, particularly the validation that polypharmacy creates compounding challenges (OR = 9.99, p = 0.024) rather than isolated problems. However, the sample was limited to English-speaking participants in rural Michigan, which may affect the generalizability of results to other geographic areas or more diverse rural populations. Even so, the accessibility barriers highlighted in this study, such as transportation challenges, limited digital literacy, and mobility issues, are widely reported across rural communities in the United States [21].

The self-paced packet model offers particular promise for public health practice, especially given our statistical evidence of its superior reach and the similar polypharmacy burden it achieved compared to in-person sessions (p = 0.766). Its cost-effectiveness makes it suitable for resource-limited rural health departments. The packet’s multi-format approach included printed materials, a USB drive with the video presentation, and links or QR codes to access content online. This variety accommodated participants with different levels of technology access and digital literacy, helping to address health equity concerns. Implementation through existing networks (senior centers, libraries, Commission on Aging offices) demonstrates scalability potential.

Future research should focus on several key methodological improvements informed by our statistical findings. Standardizing survey instruments across intervention groups would enable direct statistical comparisons while maintaining accessibility features, including validated scales for medication management self-efficacy and polypharmacy knowledge retention. Given our significant findings about the association between taking multiple medications and experiencing management difficulties (p = 0.011) and the relationship between polypharmacy status and facing multiple simultaneous medication challenges (p = 0.024), future studies should include larger sample sizes powered to detect smaller effect sizes and should investigate whether these associations hold across different populations and settings. Pre-intervention surveys that identify key barriers to medication management can help inform the most effective strategies to address them. Expanding the list of health condition categories beyond those currently included, such as adding arthritis, chronic pain, dementia, kidney disease, chronic obstructive pulmonary disease, or cardiovascular disease, would allow for more detailed analysis of how specific chronic conditions affect medication management challenges. This could also help identify patterns in how multiple conditions interact and contribute to medication complexity.

For the self-paced packet intervention, future iterations should include comprehensive printed pamphlets with large, accessible typography covering all video content to ensure complete accessibility. Video components can be enhanced through personal presenter introductions, professional voiceover narration, closed captions, and concluding segments to improve engagement.

This study focused on older adults in rural areas; however, it could be beneficial to study these interventions on older adults in urban areas as well. Expanding geographic and demographic diversity would enhance generalizability to broader populations, including non-English-speaking communities and areas with different healthcare infrastructure.

Study limitations

Several important limitations affect how these findings should be interpreted. The surveys used in the two intervention groups were not fully standardized. For example, the in-person group was asked to define polypharmacy using a multiple-choice question with several distractors, while the self-paced packet group received a simpler true or false version. Other survey items assessing knowledge and confidence also varied in format or wording across groups. These inconsistencies limited the ability to make direct statistical comparisons and may have introduced measurement bias. Additionally, our use of informal cognitive assessment rather than standardized cognitive screening tools may have resulted in the inclusion of participants with mild cognitive dysfunction, and the absence of random assignment may have led to selection bias, as participants selected their preferred format based on accessibility needs or convenience, both of which could affect the reliability and validity of our findings.

The small sample sizes, with 18 participants in the in-person group and 33 participants in the self-paced packet group, further limit statistical power and generalizability. While we achieved statistical significance for some key associations, larger samples would enable the detection of smaller effect sizes and more robust subgroup analyses. Because post-intervention data were collected immediately, the study could not assess long-term knowledge retention or behavioral change. Finally, without a control group, we cannot definitively attribute observed improvements to the interventions alone.

## Conclusions

This exploratory pilot study found that both educational interventions showed potential for improving polypharmacy knowledge and medication management confidence among rural older adults. The self-paced packet format reached more participants, included a wider geographic range, and received consistently positive feedback. Descriptive trends suggest that the packet approach may offer greater accessibility and scalability for rural populations. By offering printed materials, USB drives, and online options, the intervention addressed common barriers such as transportation challenges, limited digital literacy, and mobility concerns. Our inferential statistical analysis revealed significant associations that validate the clinical importance of addressing polypharmacy in this population. Polypharmacy was significantly associated with both individual medication management difficulties (p = 0.011) and multiple simultaneous challenges (p = 0.024, OR = 9.99), demonstrating that medication burden creates compounding rather than isolated problems. These findings support the continued development and future testing of flexible, accessible educational tools to promote medication safety among underserved older adults.
